# Molecular Basis for CCRL2 Regulation of Leukocyte Migration

**DOI:** 10.3389/fcell.2020.615031

**Published:** 2020-12-10

**Authors:** Tiziana Schioppa, Francesca Sozio, Ilaria Barbazza, Sara Scutera, Daniela Bosisio, Silvano Sozzani, Annalisa Del Prete

**Affiliations:** ^1^Department of Molecular and Translational Medicine, University of Brescia, Brescia, Italy; ^2^Humanitas Clinical and Research Center Rozzano-Milano, Rozzano, Italy; ^3^Microbiology Section, Department of Public Health and Pediatric Sciences, University of Torino, Turin, Italy; ^4^Laboratory Affiliated to Istituto Pasteur Italia-Fondazione Cenci Bolognetti, Department of Molecular Medicine, Sapienza University of Rome, Rome, Italy

**Keywords:** leukocyte recruitment, chemerin, inflammatory diseases, tumor microenvironment, atypical chemokine receptors

## Abstract

CCRL2 is a seven-transmembrane domain receptor that belongs to the chemokine receptor family. At difference from other members of this family, CCRL2 does not promote chemotaxis and shares structural features with atypical chemokine receptors (ACKRs). However, CCRL2 also differs from ACKRs since it does not bind chemokines and is devoid of scavenging functions. The only commonly recognized CCRL2 ligand is chemerin, a non-chemokine chemotactic protein. CCRL2 is expressed both by leukocytes and non-hematopoietic cells. The genetic ablation of CCRL2 has been instrumental to elucidate the role of this receptor as positive or negative regulator of inflammation. CCRL2 modulates leukocyte migration by two main mechanisms. First, when CCRL2 is expressed by barrier cells, such endothelial, and epithelial cells, it acts as a presenting molecule, contributing to the formation of a non-soluble chemotactic gradient for leukocytes expressing CMKLR1, the functional chemerin receptor. This mechanism was shown to be crucial in the induction of NK cell-dependent immune surveillance in lung cancer progression and metastasis. Second, by forming heterocomplexes with other chemokine receptors. For instance, CCRL2/CXCR2 heterodimers were shown to regulate the activation of β2-integrins in mouse neutrophils. This mini-review summarizes the current understanding of CCRL2 biology, based on experimental evidence obtained by the genetic deletion of this receptor in *in vivo* experimental models. Further studies are required to highlight the complex functional role of CCRL2 in different organs and pathological conditions.

## Introduction

Leukocyte migration is a tightly regulated process that takes place under both homeostatic and pathological conditions (David and Kubes, 2019). Chemokines control leukocyte trafficking through the interaction with their cognate receptors, belonging to the family of G protein-coupled membrane proteins (GPCRs) (Bachelerie et al., 2014; Sozzani et al., 2015; Hughes and Nibbs, 2018). A subset of proteins highly homologous to conventional chemokine receptors but unable to activate signal transduction through G proteins was identified and named Atypical Chemokine Receptors (ACKRs) (Bachelerie et al., 2014). ACKRs bind to chemokines in a rather promiscuous manner and are generally characterized by the ability to scavenge their ligands. *In vivo* evidence obtained using gene-targeted animals have highlighted the crucial role of these molecules in the negative control of inflammation (Bonecchi and Graham, 2016).

Chemokine (C–C motif) receptor-like 2 (CCRL2, also called HCR or CRAM in humans and L-CCR in mice) is a seven transmembrane receptor closely related to the chemokine receptors CCR1, CCR2, CCR3, and CCR5 (Fan et al., 1998; An et al., 2011; Del Prete et al., 2013; De Henau et al., 2016). Nevertheless, CCRL2 is unable to activate conventional G-protein dependent signaling and to induce cell directional migration, since it lacks the canonical high conserved DRYLAIV motif. Therefore, CCRL2 was originally considered a member of ACKRs family (Bondue et al., 2011). In the past few years, several ligand were proposed for CCRL2, such as CCL2, CCL5, CCL7, CCL8 (Biber et al., 2003), or CCL19 (Leick et al., 2010), but these findings were not subsequently confirmed (Zabel et al., 2008; Del Prete et al., 2013; De Henau et al., 2016). So far, the only commonly accepted CCRL2 ligand is the non-chemokine chemotactic protein chemerin (Zabel et al., 2008), a ligand shared with two other signaling receptors, namely Chemokine-Like Receptor 1 (CMKLR1) and G protein-coupled receptor 1 (GPR1) (Bondue et al., 2011; De Henau et al., 2016). Chemerin binding to CCRL2 does not induce calcium fluxes or ligand scavenging (Zabel et al., 2008; De Henau et al., 2016; Mazzotti et al., 2017) and this atypical behavior makes CCRL2 a unique member of the non-signaling GPCR chemotactic receptor family. Here we summarize the current knowledge on the expression and functions of the atypical receptor CCRL2 mostly based on the results obtained in gene targeting experiments.

## Regulation of CCRL2 Expression

In humans, two different CCRL2 splice variants are present, namely CCRL2A (or CRAM-A) coding for a 357 amino acid protein and CCRL2B (or CRAM-B) coding for a shorter receptor, lacking the first 12 amino acids in the amino-terminal domain (Yoshimura and Oppenheim, 2011; Del Prete et al., 2013). By contrast, mouse CCRL2 consists only of one single variant corresponding to CCRL2B. These orthologs are quite divergent in sequence, with only 51% identity, as compared to 80% of most of the other mouse-to-man GPCR receptor pairs (Fan et al., 1998; DeVries et al., 2006). The identity raises to 81% when considering only the first 16 amino-terminal amino acids, a short-conserved sequence that may represent the critical binding domain for chemerin (Zabel et al., 2008). However, the impact on ligand binding, if any, of the longer amino-terminal domain of CCRL2A has never been assessed. In general, only a few studies clearly indicate which variant is being investigated, or address possible differences in their expression and regulation; this is partly due to the paucity of specific reliable reagents. Nevertheless, it is becoming increasingly clear that the two isoforms may be differentially expressed and regulated: for example, CCRL2A expression is restricted to pre-B cells while other B cell-maturation stages express mainly CCRL2B (Hartmann et al., 2008). Furthermore, CCRL2A can be specifically upregulated in certain pathological conditions, such as in breast cancer by IFN-γ (Sarmadi et al., 2015). Thus, the two splice variants may possess so far unknown different biological roles and significance.

CCRL2 is expressed by cells in the hematopoietic and non-hematopoietic compartments. Among the hematopoietic cells, both CCRL2 mRNA and protein were detected in monocytes, macrophages, neutrophils, CD4 and CD8 positive T lymphocytes, B cells, monocyte-derived dendritic cells, and CD34 positive cells (Patel et al., 2001; Migeotte et al., 2002; Galligan et al., 2004; Auer et al., 2007; Hartmann et al., 2008; Catusse et al., 2010; Del Prete et al., 2013). In agreement with the first description of CCRL2 as an early LPS-inducible gene in the mouse macrophage cell line RAW264 (Shimada et al., 1998), in most of the cases, CCRL2 expression is upregulated by proinflammatory stimuli. In human monocytes, LPS alone or in combination with IFN-γ induced CCRL2 expression (Patel et al., 2001; Migeotte et al., 2002). CCRL2 mRNA was rapidly upregulated in mouse bone marrow-derived dendritic cells activated with LPS, Poly (I:C) or CD40L, reaching peak levels after 2–4 h, and decreased afterward, while CCRL2 protein levels peaked later at around 12 h and declined at the basal levels after 40 h of stimulation (Otero et al., 2010). In human neutrophils, the expression of CCRL2 was increased by proinflammatory stimuli, such as LPS or TNF-α alone or in combination with IFN-γ or GM-CSF (Galligan et al., 2004) and in neutrophils isolated from inflamed joints of arthritis patients (Auer et al., 2007). Similar CCRL2 expression kinetics was shown in mouse neutrophils (Del Prete et al., 2017). Furthermore, in mouse mast cells, CCRL2 was found to be constitutively expressed and to be further upregulated *in vitro* in BM-derived cells (Zabel et al., 2008). Also microglia and astrocytes were shown to express CCRL2 both *in vitro* and *in vivo* under inflammatory conditions (Zuurman et al., 2003; Brouwer et al., 2004). Within the non-hematopoietic compartment, CCRL2 mRNA was detected in inflamed bronchial epithelium (Oostendorp et al., 2004). Other reports described CCRL2 expression in hepatic stellate cells (Zimny et al., 2017), in adipocytes (Muruganandan et al., 2010), in skin (Banas et al., 2015) and in different cancer tissues including breast (Sarmadi et al., 2015) and prostate cancers (Reyes et al., 2017). In primary human endothelial cells, either derived from umbilical veins, dermal microvascular or brain vasculature, CCRL2 was significantly upregulated by proinflammatory stimuli (e.g., the combination of LPS, IFN-γ, and TNF-α) (Monnier et al., 2012). In endothelial cells freshly isolated from mouse lung, CCRL2 was found constitutively expressed, while in mouse liver the expression was strongly increased by inflammatory stimuli (Monnier et al., 2012). CCRL2 regulation was detected also *in vitro* in lymphatic endothelial cells stimulated with retinoid acid (Gonzalvo-Feo et al., 2014). Organ specific regulation may underscore specific functional properties of CCRL2 in different anatomical districts.

## Role of CCRL2 in the Regulation of Leukocyte Migration

A detailed analysis of CCRL2 membrane trafficking confirmed that CCRL2 efficiently binds the chemotactic protein chemerin without triggering receptor internalization, ligand scavenging, or calcium mobilization (Mazzotti et al., 2017). These results depict a unique functional profile of CCRL2 among members of non-signaling seven-transmembrane domain receptor family.

**FIGURE 1:**
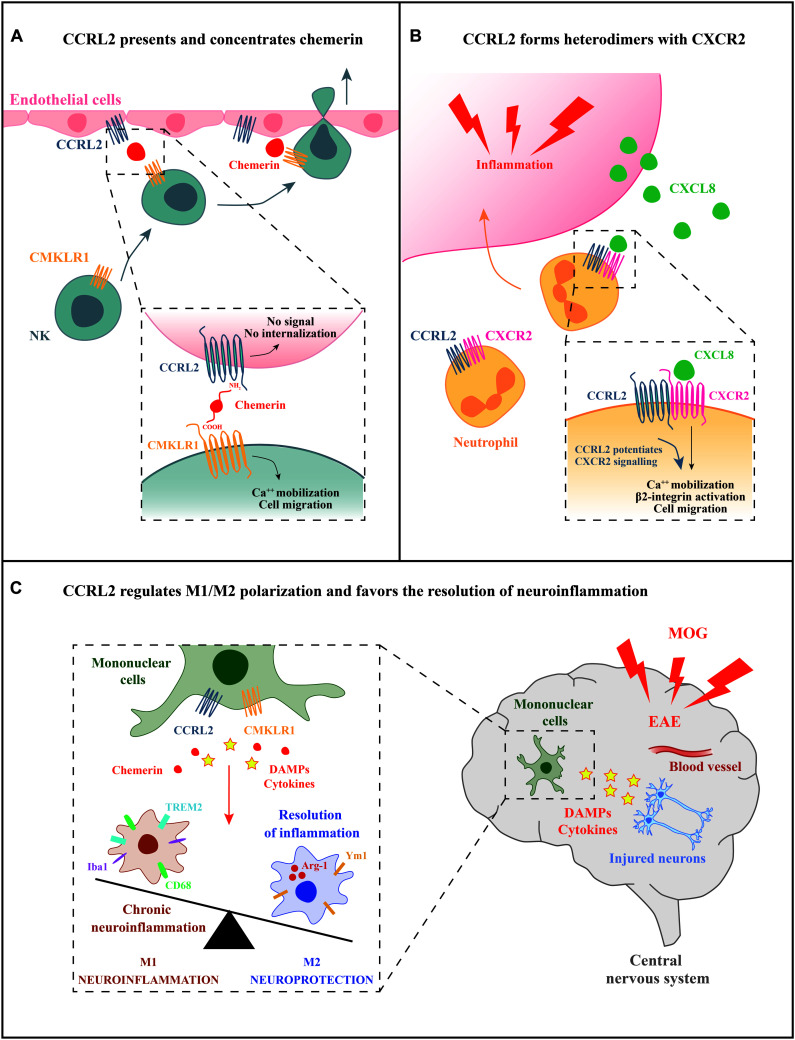
Potential mechanisms of CCRL2 regulation of leukocyte migration. **(A)** CCRL2, expressed on barrier cells, such as epithelial or endothelial cells, can act as a chemerin presenting molecule. CCRL2 binding to the N-terminus leaves chemerin C-terminus available for the interaction with CMKLR1, the functional chemerin receptor expressed by different leukocyte subsets, such as NK cells. **(B)** CCRL2 regulates CXCR2 membrane expression and integrin-mediated arrest of neutrophils on endothelial cells forming CCRL2/CXCR2 heterodimers. **(C)** CCRL2 can favor the resolution of inflammation through the regulation of M1/M2 polarization balance.

Two main functions have been described for CCRL2, both having a role in leukocyte trafficking (Figure 1). First, CCRL2, when expressed on the surface of barrier cells, such as endothelial and epithelial cells, can increase the local concentration of chemerin to form a membrane-bound chemotactic gradient for leukocytes expressing the functional chemerin receptor CMKLR1 (Zabel et al., 2008; Bondue et al., 2011; Monnier et al., 2012; Gonzalvo-Feo et al., 2014). CCRL2 binds chemerin at the N-terminus leaving the C-terminal peptide sequence accessible for the interaction with CMKLR1 (Zabel et al., 2008). By this mean, CCRL2 may promote the recruitment of CMKLR1-expressing cells, such monocytes/macrophages, dendritic cells, plasmacytoid dendritic cells, and NK cells (Del Prete et al., 2006; Sozzani et al., 2010; Tiberio et al., 2018). This CCRL2/CMKLR1 axis was shown to be active *in vivo* in the regulation of dendritic cells, mast cells, and NK cells trafficking (Parolini et al., 2007; Zabel et al., 2008; Otero et al., 2010; Monnier et al., 2012; Gonzalvo-Feo et al., 2014; Del Prete et al., 2019; Figure 1A).

A second proposed function is unrelated to the interaction with its ligand and consists in the formation of heterodimers with chemokine receptors. CCRL2/CXCR2 heterodimers were shown to represent a mechanism of fine-tuning of neutrophil migration in pathological contextures, such as inflammatory arthritis (Del Prete et al., 2017). The feature of G-protein coupled receptors to form oligomers has emerged as a physiological phenomenon that can affect several aspects of receptor functions, such as ligand targeting, signaling, and internalization properties (Mellado et al., 2001; de Poorter et al., 2013). Heterodimerization of chemokine receptors is a potential crucial step for the proper function of immune cells and represents an additional level of complexity in the promiscuous chemokine system (Mellado et al., 2001; Thelen et al., 2010; Martínez-Muñoz et al., 2018). FRET analysis revealed that CCRL2/CXCR2 heterodimers were detectable both at the cell membrane and in the cytoplasm, suggesting that the CCRL2 is involved in the intracellular retention of the CCRL2/CXCR2 heterocomplexes. Indeed, modulation of CXCR2 membrane expression by CCRL2 was shown both in transfected cells and in primary bone marrow-derived neutrophils where Ccrl2 deficiency was related with increased CXCR2 membrane expression (Del Prete et al., 2017). CCRL2 expression was also associated with increased CXCR2 signaling through ERK1/2 and small GTPases phosphorylation, and activation of β2-integrin, as detected both *in vitro* and *in vivo* by underflow and intravital microscopy (see below; Figure 1B; Del Prete et al., 2017). Collectively, these findings identify CXCR2 as a target of CCRL2 regulation. The involvement of CCRL2 in the regulation of other chemotactic receptors needs to be further explored.

## Role of CCRL2 in Inflammatory Diseases

The role of CCRL2 has emerged by the use of Ccrl2-deficient mice tested in several experimental models of inflammatory diseases (Otero et al., 2010; Gonzalvo-Feo et al., 2014; Mazzon et al., 2016; Del Prete et al., 2017). In a model of OVA-induced airways hypersensitivity, the genetic ablation of CCRL2 caused defective trafficking of antigen-loaded dendritic cells from the lung to mediastinal lymph nodes (Otero et al., 2010). In these experimental conditions, Ccrl2-deficient mice showed a protected phenotype, characterized by reduced recruitment of eosinophils and mononuclear cells to the bronchoalveolar compartment and decreased production of lung Th2 cytokines and chemokines. The defect in Th2-skewed response was directly ascribed to impaired dendritic cells migration, since it was abrogated by the intratracheal instillation of wild type dendritic cells (Otero et al., 2010). Considering the ability of CCRL2 to form heterodimers as a way to regulate chemotactic receptor function (Del Prete et al., 2017), it is possible that CCRL2 might play an unpredicted ligand-independent role in the control of pulmonary dendritic cell trafficking by the molecular interaction with CCR7, the main lymph node homing dendritic cell receptor. Similarly, CCRL2 increased tissue swelling and leukocyte infiltration in an IgE-mediated experimental model of passive cutaneous anaphylaxis (Otero et al., 2010; Gonzalvo-Feo et al., 2014; Mazzon et al., 2016; Del Prete et al., 2017). Ccrl2-deficient mice were also protected in experimental models of inflammatory arthritis. The mechanism of protection was mostly due to a defective neutrophils recruitment in the inflamed joints (Del Prete et al., 2017). The process of tissue neutrophils infiltration is implicated in the pathophysiology of rheumatoid arthritis and is controlled by a well-defined temporally and spatially cascade of chemoattractants and their cognate receptors, being the CXCL8/CXCR2 axis a major player (Chou et al., 2010; Wright et al., 2014). CCRL2 expression was described in neutrophils purified from the synovial fluid of rheumatoid arthritis patients (Galligan et al., 2004). In Ccrl2-deficient mice, CXCL8-induced neutrophils recruitment to the peritoneal cavity was found to be impaired. Similarly, neutrophils infiltration to inflamed joints was impaired in Ccrl2-deficient mice tested in collagen induced-and serum transfer induced- arthritis, two experimental models of inflammatory arthritis. In both experimental conditions, Ccrl2-deficient mice showed decreased severity of disease, lower incidence and delayed clinical onset, with reduced histopathological score. Disease protection was reversed by the adoptive transfer of CCRL2 competent neutrophils. Intravital microscopy clearly revealed that Ccrl2-deficient neutrophils displayed a strong reduction in their ability to adhere to the surface of endothelial cells in the vessels present in inflamed knee, with an increased number of rolling neutrophils on the endothelial surface. Similar results were obtained in experiments performed under flow conditions showing defective capacity of Ccrl2-deficient neutrophils to undergo rapid β2 integrin-mediated arrest in response to CXCL8. Taken together, these results support a role for CCRL2 in the regulation of CXCR2-mediated inside-out β2-integrin activation (Del Prete et al., 2017). Using different models of acute inflammation induced by zymosan and thioglycolate, Regan-Komito et al. (2017) reported that Ccrl2-deficient mice expressed an exacerbated phenotype characterized by increased neutrophils infiltration associated to increased local and systemic levels of chemerin and CXCL1. It is possible that different experimental conditions might be responsible for this apparently contrasting phenotype. Of note, the role of CCRL2 in mast cell activation *in vivo* was previously reported to be influenced by the strength of the response (Zabel et al., 2008).

A possible role for CCRL2 in the resolution phase of inflammation emerged in the chronic phase of MOG-induced experimental autoimmune encephalitis (EAE), a model that resembles the inflammatory process that characterizes multiple sclerosis (Mazzon et al., 2016; Figure 1C). In the central nervous system, CCRL2 was expressed by infiltrating mononuclear cells at the peak of clinical development of the disease. Ccrl2-deficient mice displayed increased mortality and severity of clinical score compared to control animals. In addition, the histopathological examination revealed enlarged demyelination areas and hyperactivation of microglia with unbalanced M1/M2 rate of polarization, especially during the recovery phase of the disease (Mazzon et al., 2016). Furthermore, in a model of DSS-induced colitis, chemerin has been associated with mononuclear cell polarization (Lin et al., 2014). These findings highlight a potential involvement of the chemerin/CCRL2 axis in the dynamic process of macrophage polarization, a fundamental step in the resolution of inflammation and tissue repair. Taken together, the use of mice with genetic deletion of CCRL2 has provided important insights in deciphering the molecular mechanisms of CCRL2-mediated regulation of leukocyte trafficking and pathological conditions.

## Role of CCRL2 in Tumors

CCRL2 expression was described in different cancer cells, including prostate and breast carcinoma, colorectal cancer liver metastasis and glioblastoma (Yin et al., 2012; Wang et al., 2015; Akram et al., 2016; Reyes et al., 2017). However, the functional role of CCRL2 in cancer is still unknown and needs further investigations. In NSCLC patients, elevated expression of CCRL2 was found to have a beneficial effect on overall survival and correlated with better clinical outcome, particularly at the early phase of lung tumor progression (Del Prete et al., 2019; Treeck et al., 2019).

During lung carcinogenesis CCRL2 exerts a protective role in different experimental models. Indeed, CCRL2 deficiency was associated with increased tumor burden in urethane-induced lung carcinogenesis and in a genetic model of Kras/Tp53-driven (Kras^*G*12D/+^/p53^*LoxP*^) lung tumor. Similarly, Ccrl2-deficient mice were more permissive for tumor growth following orthotopic injection of a tumor cell line obtained from Kras^*G*12D/+^/p53^*LoxP*^ mice. In all these experimental conditions, lung tumor microenvironment revealed the decrease of some myeloid cell subsets, such as monocytes, macrophages and neutrophils, and a consistent reduction of lung NK cell frequency, with the more mature NK cell subset (CD27^–^ CD11b^+^) being the most affected one. Since CCRL2 is not expressed by mouse NK cells, but was found expressed by CD31^+^ cells in the lung of tumor-bearing mice, these results further support the role of CCRL2 expression by endothelial cells in the regulation of NK cell recruitment to the lung. CCRL2 present on the surface of lung endothelial cells may act as a chemerin-presenting molecule regulating the recruitment of CMKLR1^+^ NK cells. By this mechanism, CCRL2 may shape the immune tumor microenvironment in lung cancer (Del Prete et al., 2019). This mechanism may have a more general relevance for lung tumor and metastasis (Pachynski et al., 2012). Whether the CCRL2/CMKLR1 axis is a selective pathway for the recruitment of NK cells to the lung microenvironment or rather is a pathway shared by different organs is still under investigation.

## Conclusion and Future Perspectives

Chemokines and chemotactic agonists play a crucial role in the control of leukocyte trafficking acting at different levels of regulation. Over the last few years ACKRs, a small subset of GPCRs, attracted the attention for their ability to regulate inflammatory responses. CCRL2 is closely related to ACKRs but differs from them since it does not bind chemokines or possess ligand scavenging functions (De Henau et al., 2016; Mazzotti et al., 2017). CCRL2 regulates leukocyte migration and is involved in the control of both innate and adaptive immune responses in different inflammatory diseases and cancer. Depending on the cellular context and pathological condition, CCRL2 may act as a chemerin presenting molecule or modulate the function of chemokine receptors in a ligand-independent manner. Many aspects of the biology and activity of CCRL2 remain still unexplored and need to be further elucidated. A better understanding of the precise role of this atypical receptor may pave the way toward novel and improved therapeutic strategies for the control of inflammation and tumor immune surveillance.

## Author Contributions

TS and FS conceptualized the contents. TS, FS, IB, SS, IB, DB, AD, and SS contributed to writing the manuscript. IB prepared the figure. AD and SS supervised the final version of the review manuscript. All authors contributed to the article and approved the submitted version.

## Conflict of Interest

The authors declare that the research was conducted in the absence of any commercial or financial relationships that could be construed as a potential conflict of interest.
